# Glomangiopericytoma of the Nasal Cavity with *CTNNB1* p.S37C Mutation: A Case Report and Literature Review

**DOI:** 10.1007/s12105-018-0961-z

**Published:** 2018-09-11

**Authors:** Michihisa Kono, Nobuyuki Bandoh, Ryosuke Matsuoka, Takashi Goto, Toshiaki Akahane, Yasutaka Kato, Hiroshi Nakano, Tomomi Yamaguchi, Yasuaki Harabuchi, Hiroshi Nishihara

**Affiliations:** 1 Department of Otolaryngology-Head and Neck Surgery, Hokuto Hospital, Inadacho Kisen 7-5, Obihiro, 080-0833 Japan; 2 Center for Diagnostic Pathology, Mita Hospital, International University of Health and Welfare, Tokyo, 108-8329 Japan; 3 Laboratory of Cancer Medical Science, Department of Biology and Genetics, Hokuto Hospital, Inadacho Kisen 7-5, Obihiro, 080-0833 Japan; 4 Department of Otolaryngology-Head and Neck Surgery, Asahikawa Medical University, Midorigaoka-Higashi 2-1-1-1, Asahikawa, 078-8510 Japan; 5 Keio Cancer Center, Keio University School of Medicine, 35 Shinanomachi, Shinjukuku, Tokyo, 160-8582 Japan

**Keywords:** Glomangiopericytoma (GPC), CTNNB1, Next-generation sequencing (NGS), Endoscopic sinus surgery (ESS)

## Abstract

Glomangiopericytoma (GPC) is a rare mesenchymal tumor arising from the nasal cavity or paranasal sinuses. GPC was categorized as a borderline and low-malignant-potential tumor by the World Health Organization in 2005 and accounts for less than 0.5% of all sinonasal tumors. We report a case of GPC in a 74-year-old woman with a history of recurrent epistaxis and nasal obstruction. A reddish tumor was seen in the right nasal cavity. Enhanced computed tomography showed a mass lesion occupying the right nasal cavity. The tumor, which originated from the nasal septum in the olfactory fissure area, was resected with 5-mm mucosal margins by endoscopic sinus surgery. Histologic examination revealed a uniform proliferation of oval-to-short spindle-shaped cells beneath the epithelium. Immunohistologic analysis demonstrated the tumor cells were positive for α-smooth muscle actin, β-catenin and Vimentin, and negative for AE1/AE3, Bcl-2, CD34, CD117, Factor VIIIR Ag, S-100 protein, or STAT6. The percentage of Ki-67-positive cells was approximately 5%. Genetic analysis using next-generation sequencing revealed a missense mutation in the *CTNNB1* gene (c.110C > G, p.S37C). While other *CTNNB1* mutations have been described in GPC; this is the first report of this specific mutation. The mutation was confirmed using Sanger sequencing.

**Electronic supplementary material:**

The online version of this article (10.1007/s12105-018-0961-z) contains supplementary material, which is available to authorized users.

## Introduction

Glomangiopericytoma (GPC), also called sinonasal-type hemangiopericytoma, is a rare mesenchymal tumor arising from the pericytes surrounding capillaries [1]. GPC was distinguished from hemangiopericytoma and solitary fibrous tumors and categorized as a borderline and low-malignant-potential soft-tissue tumor of the nose and paranasal sinuses by the World Health Organization in 2005 [2]. GPC accounts for less than 0.5% of all sinonasal tumors. We report a case of GPC treated with endoscopic sinus surgery (ESS) and analysis of mutations in cancer-related genes using next-generation sequencing (NGS).

## Case Report

**Fig. 1:**
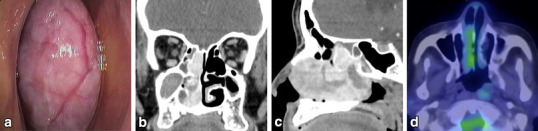
Nasal endoscopic examination showed a reddish tumor in the right nasal cavity (**a**). CT scan revealed a mass occupying the right nasal cavity, with strong enhancement (**b, c**). FDG-PET/CT showed slight uptake by the tumor (**d**)

A 74-year-old Japanese woman presented with a 1-year history of right nasal obstruction and 1-month history of epistaxis. A reddish tumor was observed in the right nasal cavity (Fig. 1a). Computed tomography (CT) scan showed a mass occupying the right nasal cavity with strong enhancement (Fig. 1b, c). FDG-PET/CT showed slight uptake by the tumor (Fig. 1d). We diagnosed a benign tumor of the nasal cavity and resected the mass by ESS under general anesthesia. The tumor, which originated from the nasal septum in the olfactory fissure area, was completely resected with 5-mm mucosa margins. The blood loss was 200 ml, and the operation time was 75 min.

**Fig. 2:**
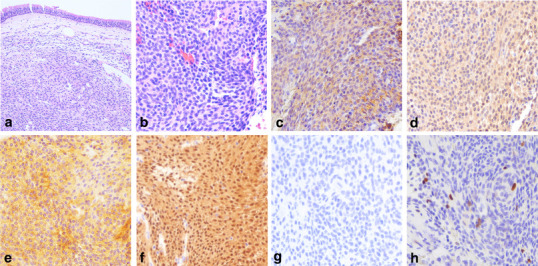
Histopathologic examination revealed that the tumor extended with a diffuse growth pattern beneath the epithelium (HE, **a**). Oval-to-short spindle-shaped cells with uniform proliferation and stromal bleeding were observed (HE, **b**). Immunohistologic analysis showed tumor cells with cytoplasmic staining for α-smooth muscle actin (SMA) (**c**) and Vimentin (**d**). Diffuse staining of tumor cells for CD99 (**e**) and nuclear staining for β-catenin (**f**) were observed. Tumor cells were not stained for STAT6 (**g**). The percentage of Ki-67-positive cells was approximately 5% (**h**). Magnification ×20 (**a**), ×400 (**b**–**h**)

Histopathologic examination revealed that the tumor extended with a diffuse growth pattern beneath the epithelium (Fig. 2a). Oval-to-short spindle-shaped cells with uniform proliferation and stromal bleeding were observed (Fig. 2b). Immunohistologic analysis showed tumor cells with cytoplasmic staining for α-smooth muscle actin (SMA) (Fig. 2c) and Vimentin (Fig. 2d). Diffuse staining for CD99 (Fig. 2e) and nuclear staining for β-catenin (Fig. 2f) were observed. Tumor cells were not stained for STAT6 (Fig. 2g), AE1/AE3, Bcl-2, CD34, CD117, Factor VIIIR Ag, or S-100 protein (data not shown). The percentage of Ki-67-positive cells was approximately 5% (Fig. 2h). On the basis of these findings, the patient was diagnosed with GPC. Two years after the surgery, the patient is alive without local recurrence or metastasis.

**Fig. 3:**
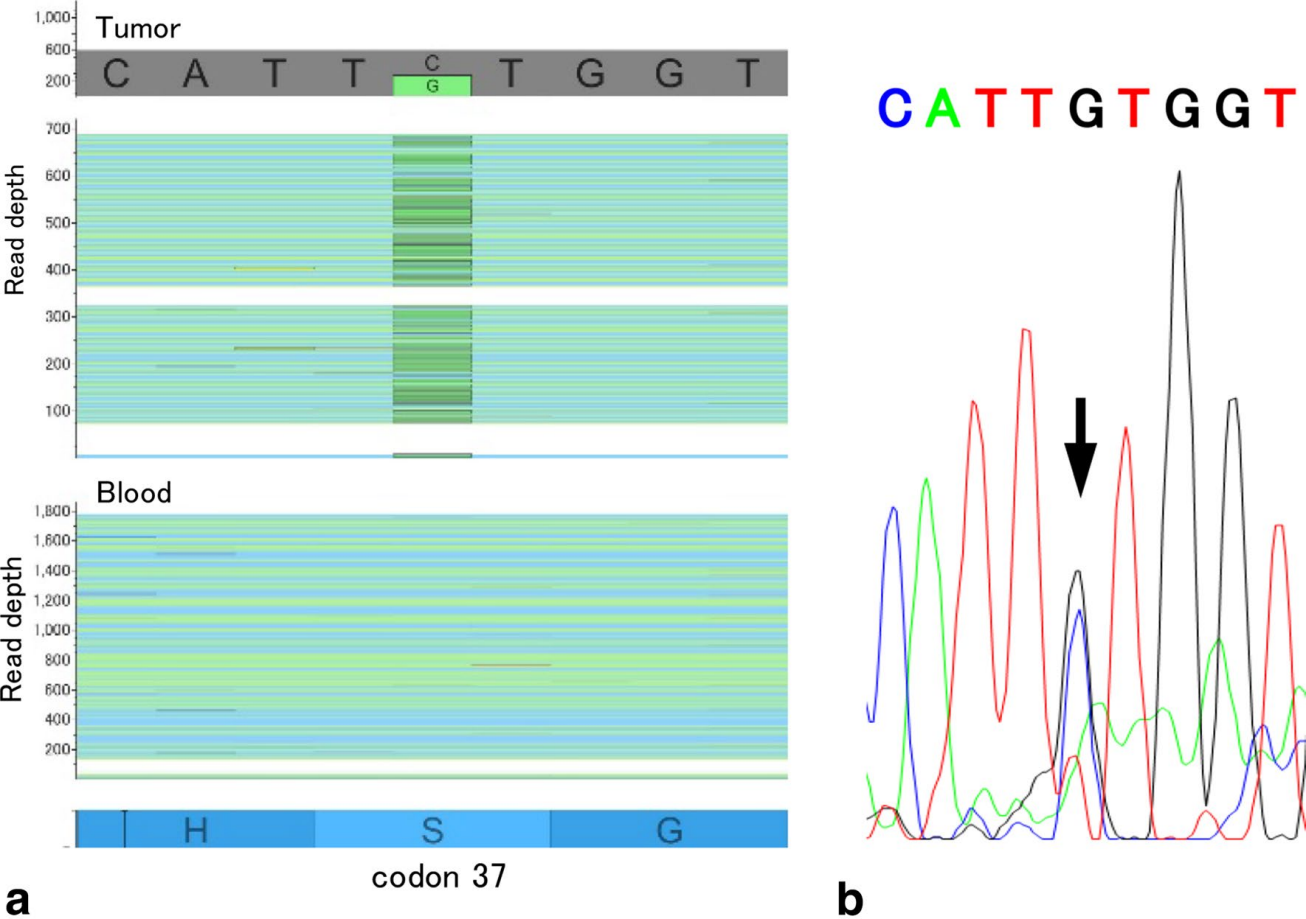
Targeted genomic DNA sequences determined using next-generation sequencing were compared between tumor and peripheral blood samples with a read depth of 700 and 1800, respectively. A *CTNNB1* mutation was identified in exon 3 with a C to G base change at nucleotide 110 (c.110C > G), leading to substitution of serine for cysteine at position 37 (p.S37C) of the protein product. The image was produced using the free software Golden Helix GenomeBrowse (http://goldenhelix.com) and modified (**a**). The mutation (c.110C > G) was confirmed using Sanger sequencing (**b**)

Genetic analysis was performed according to the manufacturer’s instructions [3]. Briefly, total DNA was extracted from 5-µm-thick formalin-fixed paraffin-embedded tissue sections of the tumor and peripheral blood samples. A GeneRead DNA seq Targeted Panel V2 human comprehensive cancer panel (NGHS-501X; Qiagen, Valencia, CA) was used for amplicon sequencing of targeted regions of 160 cancer-related genes (Table S1). Libraries were sequenced using a MiSeq (Illumina, San Diego, CA). Raw read data obtained from the amplicon sequencing were processed using online analytical resources from the GeneRead DNAseq Variant Calling Service for analysis of mutations. Among the 160 cancer-related genes examined, the analysis revealed only a missense mutation in the *CTNNB1* gene (c.110C > G, p.S37C; Fig. 3a). The mutation was confirmed using Sanger sequencing (Fig. 3b).

## Discussion

Characteristics of 23 patients with GPC identified from the literature published after 2005 are summarized in Table 1 [4–20]. Median age of patients with GPC was 60 years and ranged from 22 to 86 years. More patients were female (female: 16, male: 7). The most frequent clinical presentation was epistaxis in 18 (78%), followed by nasal obstruction in 12 (52%) and headache in 4 (17%). The tumor usually appears submucosal, beefy red, soft and hemorrhagic without surface ulceration. CT invariably shows a soft-tissue mass with strong enhancement in the unilateral nasal cavity or paranasal sinuses. Tumor limited to the unilateral nasal cavity was present in 9 (45%) patients. Nasal tumor extending to the ethmoid sinus was present in 9 (45%) patients and to the maxillary sinus in 4 (20%) of the 20 patients with detailed information. Complete surgical resection is the standard treatment in GPC because the tumor is relatively resistant to chemotherapy and radiation [7]. Surgical resection by ESS was performed in 13 (57%) of the 23 patients. Based on tumor extension, partial resection of the maxilla [9] or external incisional surgery has been reported [4–6]. We performed ESS with 5-mm safety margins for the basal part of the tumor. Recurrence after resection has been reported in 15.1% of cases, and this can be managed by additional surgery [1]. The prognosis of GPC is usually favorable (5-year overall survival rate: 88%); however, long-term follow-up is essential for the management of GPC [1].

**Table 1:** Characteristics of glomangiopericytoma patients reported in the literature published after 2005

Author	Age	Gender	Symptom	Location	Surgery	Ki-67 (%)
Angouridakis et al. [10]	45	M	Epistaxis, obstruction	N, E, M, S	ESS	
Worden et al. [11]	78	F	Epistaxis, obstruction, rhinorrhea, headache	N	ESS	
Dandekar and McHugh [4]	48	F	Epistaxis, obstruction	N, E	Medial maxillectomy	
Higashi et al. [12]	60	M	Epistaxis, obstruction	N	ESS	
Oosthuizen et al. [14]	32	F	Epistaxis, obstruction, anosmia, headache, proptosis	N, E, S, A	ESS	< 1
Arpaci et al. [13]	68	F	Obstruction, headache, hyposmia	N	ESS	
Jung et al. [5]	42	F	Epistaxis	N, E, O	ESS + External incision	
Verim et al. [16]	72	F	Epistaxis, obstruction	N	ESS	
Lee et al. [15]	60	F	Osteomalacia	M	Caldwell-Luc	
Gokdogan et al. [17]	32	M	Epistaxis, obstruction	N, E, M, S	ESS	
Roy et al. [18]	60	F	Epistaxis, obstruction	N, E	ESS	
Handra-Luca et al. [22]	86	F	Obstruction	N	Resection	5
Zielinska-Kazmiersk et al. [5]	80	M	Epistaxis, obstruction	N, M (bil)	External incision	2
Psoma et al. [7]	55	M	Obstruction	N, E	ESS	
Oliveira et al. [19]	60	F	Epistaxis, obstruction	N, E, O, A	ESS	
Kim et al. [20]	82	F	Epistaxis, rhinorrhea, headache	N	ESS	
Kim et al. [20]	57	F	Pain	N	Resection	
Anzai et al. [8]	68	M	Epistaxis	N	ESS	< 5
Al Saad et al. [9]	22	F	Epistaxis	N, M, H	Partial resection of maxilla	
Suzuki et al. [24]	81	M	Epistaxis	Unknown	Resection	< 1
Suzuki et al. [24]	62	F	Epistaxis	Unknown	Resection	1
Suzuki et al. [24]	81	F	Epistaxis	Unknown	Resection	2
Present case	74	F	Epistaxis, obstruction	N	ESS	5

*N* nasal cavity, *E* ethmoid sinus, *M* maxillary sinus, *S* sphenoid sinus, *O* orbita, *A* anterior skull base, *H* hard palate, *bil* bilateral

**Fig. 4:**
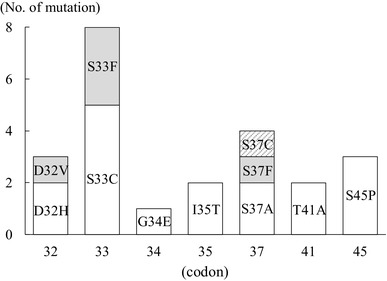
Twenty-three reported types of mutation in the *CTNNB1* gene in cases of glomangiopericytoma as reported by Lacosta et al. [21], Haller et al. [23], Anzai et al. [8], and Suzuki et al. [24]. Mutations were combined with that identified in the present case and shown as a bar graph. One patient reported by Haller et al. harbored two mutations (p.G34E and p.S37F)

GPC is diagnosed by characteristic histology showing epithelioid cells in a perivascular pattern with frequent perivascular hyalinization. Tumor cells are immunohistologically positive for cytoplasmic SMA and Vimentin, and nuclear β-catenin in 80–100%. Tumor cells exhibit no strong diffuse staining for CD34 and are basically negative for AE1/AE3, Bcl-2, CD34, CD99, CD117, Factor VIIIR Ag, S-100 protein, and STAT6 [1, 21]. Some reports demonstrated that tumor cells were positive for CD99 in agreement with our result [22]. High Ki-67 index (> 10%) is a prognostic factor for aggressive behavior [1, 21], although 5% was the highest index in the 8 patients analyzed (Table 1). Nuclear staining for β-catenin is reported to be a diagnostic marker of GPC [21, 23]; however, there are only 4 reports describing mutations in the *CTNNB1* gene as well as nuclear β-catenin expression in GPC [8, 21, 23, 24]. Mutations in the amino-terminal region of *CTNNB1* gene, which encodes β-catenin, activate the Wnt-signaling pathway. After activation, β-catenin is stabilized by phosphorylation and translocates to the nucleus. The accumulation of β-catenin in the nucleus activates transcriptional factors, promoting tumorigenesis and proliferation of tumor cells [23]. To date, 23 types of mutation in the *CTNNB1* gene in GPC have been described (Fig. 4) [8, 21, 23, 24]. All of the *CTNNB1* mutations, including that identified in the present case, involve a single-base substitution in exon 3. Eight (35%) of the 23 mutation types occur at codon 33. Four mutation types (19%) occur at codon 37, including 2 p.S37A, 1 p.S37F, and 1 p.S37C mutation. The *CTNNB1* p.S37C (c.110C > G) mutation we detected has not been observed in previous reports regarding GPC. The p.S37C mutation accounts for only 2.8% of all 6939 *CTNNB1* mutations analyzed in various mesenchymal and epithelial neoplasms, including hepatocellular, endometrial, ovarian, and pituitary tumors, according to COSMIC (April 2018). NGS is an increasingly important method for detecting mutations in cancer-related genes, as it can be used to simultaneously test for multiple mutations of interest in a short period. Targeted NGS is more cost efficient and faster than previous sequencing methods. We analyzed 160 cancer-related genes for mutations in just 2 days using NGS and detected only one mutation in the *CTNNB1* gene.

Differential diagnosis of GPC should be made to distinguish the tumor from solitary fibrous tumors (hemangiopericytoma), glomus tumors, desmoid-type fibromatosis, and nasopharyngeal angiofibroma [2]. Solitary fibrous tumors are characterized by chromosomal translocation resulting in the formation of a *NAB2-STAT6* fusion gene. Nuclear staining of STAT6 resulting from the translocation of the NAB2-STAT6 fusion protein to the nucleus is a gold-standard marker for the tumor [8]. GPC is immunohistologically negative for STAT6 and NAB2-STAT6 fusion gene transcripts [25]. Glomus tumors and GPC exhibit many histologic and immunohistologic similarities, including the perivascular histologic pattern and SMA expression [1]. However, glomus tumors lack β-catenin nuclear expression and *CTNNB1* mutations [26]. Fibroblastic neoplasms such as nasopharyngeal angiofibroma and desmoid-type fibromatosis exhibit nuclear β-catenin expression and *CTNNB1* mutations; however, these tumors are histologically different from GPC [21]. Thus, genetic analysis of oncogenes by NGS is useful for distinguishing vascular neoplasms originating from the head and neck region.

## Conclusion

We reported a rare case of GPC successfully treated with ESS. A *CTNNB1* p.S37C mutation was detected by NGS and Sanger sequencing methods, and it was the first report of this specific mutation.

## Electronic supplementary material

Below is the link to the electronic supplementary material.

Supplementary material 1 (XLSX 11 KB)
